# SGLT2 Inhibitors in Patients with Urogenital Malformations and Urinary Diversions: Risks, Benefits, and Clinical Considerations

**DOI:** 10.3390/medicina61050921

**Published:** 2025-05-20

**Authors:** Mohammed Abdulrasak, Ali Someili, Mostafa Mohrag

**Affiliations:** 1 Department of Clinical Sciences, Lund University, 22100 Malmo, Sweden; 2 Department of Gastroenterology and Nutrition, Skane University Hospital, 20502 Malmo, Sweden; 3 Department of Medicine, Faculty of Medicine, Jazan University, Jazan 45142, Saudi Arabia; asomeili@jazanu.edu.sa (A.S.); mmohrag@jazanu.edu.sa (M.M.)

**Keywords:** SGLT2 inhibitors, urogenital malformations, urinary tract infections, urinary diversions, benign prostatic hyperplasia

## Abstract

*Background*: Sodium-glucose cotransporter-2 inhibitors (SGLT2i) are increasingly used in patients with type 2 diabetes, chronic kidney disease, and heart failure. However, their safety and efficacy in patients with congenital or surgically altered urogenital anatomy remains underexplored. *Methods*: We conducted a narrative review of current evidence regarding the use of SGLT2i in patients with urinary tract malformations, urinary diversions, and functional voiding disorders. Key risks, clinical considerations, and management strategies were synthesized from the existing literature and case reports. *Results*: Patients with benign prostatic hyperplasia, vesicoureteral reflux, neurogenic bladder, nephrostomies, and ileal conduits may face increased risks of urinary tract infections, fungal colonization, and therapy-related complications due to persistent glycosuria and altered urinary flow. Nevertheless, these patients may still benefit from SGLT2i’s systemic renal and cardiovascular effects. Individualized risk assessment, close monitoring, and multidisciplinary management are essential. *Conclusions*: Patients with urological abnormalities represent a high-risk but potentially high-reward population for SGLT2i therapy. A cautious, tailored approach is necessary, and future dedicated research is urgently needed to better guide clinical practice.

## 1. Introduction

The introduction of sodium-glucose cotransporter-2-inhibitors (SGLT2i) has positively affected the outcomes of patients suffering from type 2 diabetes (T2D) [1,2], chronic kidney disease (CKD) [3], and congestive heart failure (CHF) [4,5]. This is mainly through the drug’s ultimately glucosouric effect which improves outcomes for the aforementioned patient groups [6]. Large, randomized trials [7] alongside real-world data [8,9,10] have cemented SGLT2i role in reducing CHF admissions [11,12], slowing CKD progression [13,14] and lowering cardiac mortality [15,16], whereby current guidelines have—for the relevant indications—included SGLT2i as a cornerstone in the treatment of these conditions [17,18,19].

While SGLT2i benefits are well demonstrated, their safety in patients with abnormal genitourinary anatomy is less well studied [20]. These individuals include patients with congenital deformities including vesicourethral reflux, neurogenic bladder, and bladder exstrophy, alongside those with other aberrancies including benign prostate hyperplasia (BPH), chronic nephrostomies or ileal deviations. Such urological pathologies often lead to exclusion from different studies with regard to SGLT2i-related outcomes [21,22]. These patients have a generally higher rate of bacterial and fungal UTIs given the altered urinary flow mechanics [20,23,24].

Recent studies have shown interest in exploring the risks of genitourinary infections in these select high-risk groups [20]. This risk appears particularly elevated in elderly individuals, females, and those with poorly controlled diabetes, and has been associated with early discontinuation of SGLT2i therapy in real-world settings [25]. In spite of this, there remains a gap in the clinical literature regarding patients with structural or surgically modified urinary tracts and SGLT2i usage. These unique anatomical and physiological contexts may influence not only infection risk but also drug efficacy and tolerability and are often underrepresented in clinical trials [26].

This narrative, non-systematic review aims to synthesize current evidence related to the use of SGLT2i in patients with urogenital malformations and urinary diversions, based on the sparsely available literature. We highlight the specific risks, clinical challenges, and potential benefits in this understudied population, offering guidance for individualized patient care and identifying areas for future research.

The literature search was conducted using PubMed, Scopus, and Google scholar databases up to April 2025. Keywords included combinations of “SGLT2 inhibitors”, “urinary tract infections”, “urogenital malformations”, “urinary diversion”, “benign prostatic hyperplasia”, and “neurogenic bladder”. Studies included peer-reviewed original articles, case reports, clinical guidelines, and reviews written in English. We prioritized papers focusing on anatomical urinary abnormalities and SGLT2i safety or efficacy.

## 2. Mechanism of SGLT2 Inhibitors and Genitourinary Implications

SGLT2i exert their effect through blocking glucose reabsorption in the proximal convoluted tubule of the nephron [27]. This results in glycosuria, in addition to natriuresis and osmotic diuresis, which contributes to reduced glycemia, reduced volume overload, and reduction in blood pressure [28,29]. In addition, there is an intra-glomerular effect whereby there is an increased afferent tone and decreased efferent tone which reduces the intraglomerular pressure and thereby reducing the risk for hyperfiltration injury, slowing down CKD progression [30,31].

In spite of this, the persistent glycosuria may predispose to certain complications, mainly infections. Both fungi and bacteria prosper in a glucosuric environment, which has been shown to be especially true for fungal infections [20,32,33] but not as clear for bacterial ones in the setting of SGLT2i use [34]. The risk is further elevated in patients with abnormal urinary tract anatomy or function, given that urinary stasis or poor urinary drainage may further prolong the exposure of glucose-rich urine to the urinary tracts [35].

Furthermore, SGLT2i—given their diuretic effect—may increase urinary frequency, urgency, and dribbling symptoms, which may pose a burden in patients with anatomical or functional voiding issues, including those with BPH and neurogenic bladder [36,37,38,39]. In such settings, incomplete emptying or prolonged urine contact with mucosal surfaces may increase susceptibility to ascending infections or mucosal irritation.

## 3. Patients with Urinary Tract Abnormalities: Clinical and Safety Considerations

In clinical practice, a significant portion of adults present with urinary tract abnormalities—either functional or structural—that may increase the risk of complications when SGLT2i are prescribed [20]. These include common conditions such as BPH, which is itself a notable contributor to early SGLT2i discontinuation given the predisposition for those patients to frequent urination [40]. Less common but clinically relevant abnormalities include vesicoureteral reflux, neurogenic bladder, and various forms of surgically altered urinary anatomy.

BPH remains the most frequent cause of lower urinary tract symptoms (LUTS) in adult males and is highly prevalent among patients with CKD and T2D [41,42,43], both of which are key indications for SGLT2i therapy. BPH can cause bladder outlet obstruction, impairing bladder emptying and leading to urinary retention and stasis, which in turn increases the likelihood of development of UTIs [44,45]. This risk is particularly pronounced in patients receiving SGLT2i, where persistent glycosuria provides an additional substrate for microbial growth. Furthermore, a subset of BPH patients may rely on intermittent or indwelling catheterization, compounding their infection risk [46,47].

Patients with prior urological surgery—including nephrostomy placement, ileal conduit diversion, or bladder augmentation—present with anatomical modifications that further alter urinary flow and exposure excreted glucose in the urine [48,49]. Case reports have described increased rates of both bacterial and fungal infections in these individuals, as well as potential loss of drug efficacy in ileal conduit patients, where glucose may be reabsorbed through intestinal SGLT transporters. Additionally, worsening metabolic acidosis has been observed in patients with ileal-based diversions, who may already have a predisposition due to their altered mucosal and renal handling of solutes [50,51,52].

While these structural and functional alterations vary in origin, they converge on a shared clinical challenge, namely the increased susceptibility to urological infections in the setting of pharmacologically induced glycosuria. Recognizing this risk is essential when evaluating patients for SGLT2i therapy. For those with BPH-related urinary retention or surgically altered systems, a detailed urological history, assessment of voiding function, and individualized risk-benefit analysis should precede initiation of treatment [40].

## 4. Potential Benefits of SGLT2 Inhibitors in These Populations

Despite concerns regarding genitourinary adverse effects, many patients with urinary tract abnormalities are also among those who stand to benefit the most from SGLT2i therapy. Individuals with CKD, T2D and cardiovascular comorbidities—conditions that commonly coexist with BPH, neurogenic bladder, or a history of urological reconstruction—may experience meaningful systemic improvements [53,54,55].

The renal benefits of SGLT2i, including reduction in albuminuria and attenuation of eGFR decline, may be especially valuable in patients with congenital or acquired anatomical abnormalities that predispose to progressive renal damage [56]. These benefits are not dependent on glycemic status and extend to patients with non-diabetic CKD, a population that overlaps significantly with those affected by recurrent infections, high intravesical pressures, or surgical urinary alterations [57,58].

Moreover, the cardioprotective effects of SGLT2i—including reductions in heart failure hospitalization [59] and cardiovascular mortality—remain a key justification for their use [60], even in patients with complex urological histories. This is particularly relevant in older adults with BPH, who often present concomitantly with a constellation of metabolic and cardiovascular risk factors in addition to their BPH [61,62].

While caution is warranted, the presence of a urological abnormality should not lead to automatic exclusion from therapy. Rather, appropriate patient selection, individualized risk assessment, and careful monitoring can allow for safe and effective use of SGLT2i in this population. In those with stable urological status and no recent history of severe infection, the balance of benefit versus risk may still favor treatment initiation.

## 5. Special Considerations: Post-Surgical and High-Risk Anatomies

Patients with post-surgical urinary tract alterations represent a particularly vulnerable and underexplored subgroup. Those with ileal conduit diversions, continent urinary reservoirs, or nephrostomy tubes often face chronic colonization [63,64], non-physiological urinary flow dynamics [65,66], and exposure of non-native mucosal tissue to glucose-rich urine [35]. Case reports have described instances of therapy failure due to glucose reabsorption in bowel-derived conduits, as well as severe infections and metabolic complications such as hyperchloremic acidosis in this setting [49,50,51].

Similarly, patients with nephrostomy-dependent drainage or those requiring chronic catheterization may face a compounded risk for infection, particularly when glycosuria promotes local fungal overgrowth or supports biofilm development [67,68]. These individuals require close collaboration between nephrology, urology, and primary care to determine the safety and timing of SGLT2i use, with some cases warranting either deferral or more intensive monitoring if therapy is pursued.

In the immediate postoperative setting—such as following bladder reconstruction, stent placement, or augmentation cystoplasty—temporary avoidance of SGLT2i therapy may be warranted until urinary flow is re-established and the risk of infection is reduced [69]. This approach mirrors current practice in other high-infection-risk states, such as active diabetic foot disease, where SGLT2i are commonly withheld to prevent exacerbation of ongoing infection or risk of sepsis [19]. In addition to this, and although being rare, euglycemic diabetic ketoacidosis has been reported with SGLT2i, particularly during acute illness, perioperative periods, or in patients with prolonged fasting [70].

Reinitiation of SGLT2i can be considered once healing has progressed and the anatomical situation has stabilized. Ultimately, individualization of therapy based on anatomical factors, recovery status, and infection history is essential for the safe use of SGLT2i in this population.

The following table (Table 1) provides a summary of urological conditions commonly encountered in clinical practice and outlines specific risk factors and suggested considerations when initiating SGLT2i therapy in each context. This overview may serve as a practical guide for clinicians managing patients with altered urinary tract anatomy.

## 6. Clinical Recommendations

When considering the use of SGLT2i in patients with structural or functional urinary tract abnormalities, a cautious and individualized approach is essential. This is also highly relevant given that current major guidelines—including KDIGO and the UK Kidney Association—do not specifically address patients with congenital or surgically altered urinary tracts in their recommendations for SGLT2i use [19,71]. Clinicians should begin with a detailed urological history, assessing factors such as recurrent urinary tract infections (UTIs), voiding dysfunction, catheter use, prior urinary diversion surgery, and known anatomical abnormalities [45]. Repeated infections in these patients may not only lead to short-term morbidity but also contribute to cumulative kidney injury and long-term risk of chronic kidney disease progression [72]. Patients can be loosely stratified into low- or high-risk categories based on these features.

In lower-risk individuals—those with intact urinary anatomy, no recent infections, and normal bladder function—SGLT2i may generally be initiated safely, with routine monitoring [73]. Conversely, higher-risk patients, including those with neurogenic bladder, BPH with significant post-void residuals, nephrostomies, or ileal conduits, require more careful evaluation [45]. In these cases, multidisciplinary input from urology and nephrology may be warranted, particularly if glycosuria is expected to exacerbate colonization or infection risk [74].

In patients with known urological abnormalities, a baseline assessment of renal function, urinalysis, and voiding pattern (including post-void residual if applicable) is recommended before initiating therapy [19,36]. A follow-up visit within 2–4 weeks of starting SGLT2i may be considered to assess for early signs of infection or urinary retention, in addition to a reassessment of the renal function 2 weeks after initiation of therapy [18,19,36,71]. For those with high infection risk, additional safety measures such as increased hydration, hygiene education, or coordination with urology may be beneficial [22,74].

Delaying therapy initiation following recent urological surgery or active infection is often advisable, mirroring recommendations seen in diabetic foot disease management [19,69]. For patients in whom SGLT2i therapy is initiated, close follow-up is necessary, with clear patient education on signs of genitourinary infections, proper hygiene, and the importance of reporting early symptoms of potential UTI [75]. Prompt discontinuation should be considered in the setting of systemic infection or intolerance. This individualized and collaborative approach allows for maximization of the systemic benefits of SGLT2i while mitigating risks in anatomically or functionally complex patients.

## 7. Future Directions

Despite the growing clinical use of SGLT2i across a broad range of indications, significant gaps remain regarding their safety and efficacy in patients with congenital, acquired, or surgically modified urological anatomy or individuals with high-risk for UTI development. This patient population is routinely excluded from randomized controlled trials [76,77], leading to an evidence void in precisely the individuals who may face the greatest risk of genitourinary complications from therapy. Future research efforts must address this gap to inform clinical decision-making and improve outcomes.

First and foremost, prospective observational studies and clinical trials specifically enrolling patients with urological abnormalities are urgently needed. Populations of interest include those with vesicoureteral reflux, neurogenic bladder, ileal conduits, bladder augmentations, chronic catheterization, and nephrostomy drainage. Such studies should be designed to assess the incidence of UTIs, genital mycotic infections, and urosepsis, as well as broader outcomes such as renal function preservation and cardiovascular benefits [78,79,80]. Establishing whether these patients derive similar systemic benefits to those observed in broader populations, or whether infection risks significantly outweigh advantages, is critical.

Second, there is a need for pharmacokinetic and pharmacodynamic studies specifically evaluating SGLT2i in patients with urinary diversions involving bowel segments. The possibility of glucose reabsorption through intestinal SGLT transporters raises questions regarding both therapeutic efficacy and unexpected adverse effects, such as worsened metabolic acidosis [51]. Understanding how altered urinary tract anatomy influences drug handling, glucose kinetics, and local uroepithelial exposure would provide mechanistic insights necessary for safer prescribing.

In addition, biomarker development and validation should be prioritized. Identifying laboratory or imaging markers that predict infection susceptibility or therapy failure in patients with anatomical urinary tract abnormalities could facilitate better patient selection and individualized therapy decisions. Biomarkers might include inflammatory cytokine profiles, urinary tract colonization patterns, or imaging-based measures of urinary flow and residual volumes.

Integration of patients with complex urinary tract anatomy into post-marketing surveillance programs and real-world data registries is another critical step. Large registries could capture adverse events and treatment outcomes over time, helping generate hypotheses for future randomized controlled trials and allowing for better risk modeling.

Moreover, guidelines and expert consensus statements should be updated to include considerations for these special populations. Current clinical recommendations largely reflect extrapolation from broader diabetic and CKD cohorts, but emerging real-world evidence could allow for more nuanced risk stratification and monitoring protocols. Finally, qualitative research exploring patient-reported experiences and adherence challenges in this subgroup may reveal additional barriers to successful therapy and highlight areas for patient education improvement.

This review is limited by the relatively small number of published studies specifically addressing SGLT2i use in patients with altered urogenital anatomy. Much of the available data are derived from case reports or subgroup analyses, and patient populations remain heterogeneous. As such, our conclusions should be interpreted cautiously and further dedicated research is warranted.

## 8. Conclusions

SGLT2i offer significant cardio-renal benefits in patients with type 2 diabetes, chronic kidney disease, and heart failure. However, their use in individuals with congenital, acquired, or surgically altered urogenital anatomy presents unique clinical challenges due to altered urinary dynamics, impaired drainage, and increased infection susceptibility. Factors such as persistent glycosuria, urinary stasis, and catheter- or stoma-related colonization further compound these risks.

Despite these concerns, many patients with structural or functional urinary tract abnormalities may still derive meaningful benefit from SGLT2i therapy. To optimize outcomes, clinicians should perform individualized risk assessments prior to initiation, including a review of urinary symptoms, infection history, and urological interventions. Baseline renal function and urine studies may aid in establishing risk, and early follow-up is advisable in high-risk cases.

Scenarios warranting extra caution or urology referral may include patients with indwelling catheters, ileal conduits, recurrent UTIs, or significant post-void residuals. In these populations, a multidisciplinary approach is essential to balance potential benefits with infection risks.

Future research must prioritize these underrepresented groups through targeted trials, biomarker validation, and enhanced pharmacovigilance. Until such data are available, a cautious yet proactive approach remains the safest path toward integrating SGLT2i into the care of patients with complex urogenital anatomies.

## Figures and Tables

**Table 1:** Summary of relevant urological conditions with main risk factors for UTIs and clinical considerations.

Condition	Key Risk Factors	Clinical Considerations
Benign Prostatic Hyperplasia (BPH)	Urinary retention, stasis, catheterization	Assess voiding efficiency before initiation; monitor for UTIs; counsel on hygiene
Neurogenic Bladder	Incomplete emptying, high post-void residuals, catheter use	Evaluate bladder function; consider urology referral; increased infection monitoring
Vesicoureteral Reflux (VUR)	Retrograde urine flow, chronic colonization	Higher risk of ascending infections; initiate SGLT2i cautiously if VUR active
Ileal Conduit/Urinary Diversion	Exposure of bowel mucosa to glycosuria, metabolic acidosis, infection risk	Monitor for fungal/bacterial infections; be aware of possible glucose reabsorption
Nephrostomy Tube	Chronic drainage, biofilm formation, fungal colonization	Intensive infection surveillance; consider deferring initiation if active colonization present

## Data Availability

The data presented in this publication are available within the text of the manuscript.
